# Improved Decision-Making Confidence Using Item-Based Pharmacometric Model: Illustration with a Phase II Placebo-Controlled Trial

**DOI:** 10.1208/s12248-021-00600-1

**Published:** 2021-06-02

**Authors:** Carolina Llanos-Paez, Claire Ambery, Shuying Yang, Maggie Tabberer, Misba Beerahee, Elodie L. Plan, Mats O. Karlsson

**Affiliations:** 1 Department of Pharmacy, Uppsala University, Box 580, 751 23 Uppsala, Sweden; 2 Clinical Pharmacology Modelling and Simulation, GlaxoSmithKline plc, London, UK; 3 Patient Centred Outcomes: Value Evidence and Outcomes, GlaxoSmithKline plc, Brentford, Middlesex, UK

**Keywords:** EXACT, Item response theory, Mixed-effects model repeated measures, Non-linear-mixed-effects models, Power comparison

## Abstract

This study aimed to illustrate how a new methodology to assess clinical trial outcome measures using a longitudinal item response theory–based model (IRM) could serve as an alternative to mixed model repeated measures (MMRM). Data from the EXACT (Exacerbation of chronic pulmonary disease tool) which is used to capture frequency, severity, and duration of exacerbations in COPD were analyzed using an IRM. The IRM included a graded response model characterizing item parameters and functions describing symptom-time course. Total scores were simulated (month 12) using uncertainty in parameter estimates. The 50th (2.5th, 97.5th) percentiles of the resulting simulated differences in average total score (drug minus placebo) represented the estimated drug effect (95%CI), which was compared with published MMRM results. Furthermore, differences in sample size, sensitivity, specificity, and type I and II errors between approaches were explored. Patients received either oral danirixin 75 mg twice daily (*n* = 45) or placebo (*n* = 48) on top of standard of care over 52 weeks. A step function best described the COPD symptoms-time course in both trial arms. The IRM improved precision of the estimated drug effect compared to MMRM, resulting in a sample size of 2.5 times larger for the MMRM analysis to achieve the IRM precision. The IRM showed a higher probability of a positive predictive value (34%) than MMRM (22%). An item model–based analysis data gave more precise estimates of drug effect than MMRM analysis for the same endpoint in this one case study.

**Supplementary Information:**

The online version contains supplementary material available at 10.1208/s12248-021-00600-1.

## INTRODUCTION

Confidence in clinical trial decision-making will depend on the precision of the outcome measures. These decisions may pan from stop/go to dose selection and usually rely on predefined targets. While sample size and collection timing may influence the outcome precision, the choice of the endpoint and the analytic methodology employed certainly is critical.

Patient-reported outcomes (PROs) are a type of clinical endpoint measure that is increasingly used in drug development not only to record how well a disease is managed from the patient’s point of view but also to provide information supporting the patient experience for inclusion within a product licence (1). These measurements are reported directly by the patient, without interpretation by a health professional (2). An example of a PRO instrument in the respiratory area is EXACT (Exacerbations of Chronic Obstructive Pulmonary Disease [COPD] Tool) (3), which consists of 14 questions related to COPD symptoms that are recorded daily using an electronic diary. The E-RS:COPD (Evaluating Respiratory Symptoms in COPD) consists of 11 items from this 14-item EXACT instrument, and it is intended to capture information specifically related to respiratory symptoms.

PROs collected using the EXACT and E-RS:COPD have been used as co-primary and secondary endpoints to assess drug effect in COPD in phase II and phase III randomized clinical trials (4–8). For E-RS:COPD, some of these trials (6, 7) have anticipated that a reduction of two points or more in the total score is indicative of a clinically meaningful improvement in symptoms. This value was obtained after assessing the performance of E-RS:COPD in three clinical trials (4). The first trial of clinical efficacy (usually phase II) is often associated with a stop/go decision. Although approaches to such decision-making may vary, the confidence interval (CI) of the drug effect estimate is typically the main component, as it shows the degree of (un)certainty related to an estimate (e.g. mean difference between treatment arms) (9). Recently, different approaches under the CI framework have been compared which can be more or less conservative depending on the criteria used (9).

The magnitude, precision, and bias of the drug effect estimate will depend on the statistical method used to analyze the data. In clinical trials, in the presence of daily observations in the same subject, the degree of correlation between observations should be taken into account. To analyze clinical trial repeated measurements that may contain ignorable missing data (missing at random or missing completely at random), the likelihood-based mixed model repeated measures (MMRM) approach has been used (10). This MMRM method has become the standard approach to analyze longitudinal data since it shows better type I error control compared to other methods such as analysis of covariance (ANCOVA) using the last observation carried forward approach to impute missing values (10, 11). MMRM analysis has been applied to analyze EXACT PRO data from a phase II clinical trial where the efficacy of danirixin was assessed in patients with COPD. Danirixin is a selective and reversible antagonist of the C-X-C chemokine receptor 2 (CXCR2) (12) and demonstrated dose-dependent inhibition of CXCL1-induced CD11b expression following single doses between 25 and 200 mg. Although danirixin showed a trend for improved respiratory and health status in patients with COPD (7), the absence of a clear efficacy benefit has been confirmed in a larger clinical trial (13).

An item response model (IRM) is an alternative longitudinal non-linear mixed-effects model (NLME) analysis approach. Following item response theory (IRT), it utilizes all components of the composite observations, which may increase the statistical power and precision to detect a drug effect (14). Furthermore, IRMs offer increased insight into the scale, by relating its items to an underlying disease state that varies among individuals and changes with time. It is worth noting that, like other recently developed PRO tools, the final 14-item EXACT was derived through the application of item analysis and Rasch analysis (15). These methods are used to ensure that the final PRO tool measures a coherent underlying disease concept across the range of disease severities. Recently, an IRM in COPD patients receiving standard of care described how to handle correlated observations by linking the IRM to a continuous-time Markov model (16).

The performance of MMRM and NLME to assess clinical efficacy has been already compared in the context of simulated paediatric diabetes trials (17); however, such comparisons have not been applied to an IRM and observed trial data. This study aims to illustrate how a new methodology to assess clinical trial outcome measures using a NLME analysis based on item-level data (IRM) could potentially replace the standard MMRM analysis of total score data.

## METHODS

### Data and Patients

EXACT data from a phase II, randomized, placebo-controlled study (NCT02130193) to investigate the safety, tolerability, pharmacokinetics (PK), pharmacodynamics, and clinical efficacy of oral danirixin in symptomatic COPD subjects were included in this analysis (7, 18). Patients received either placebo or danirixin on top of the subject’s current standard-of-care treatment for 52 weeks. To ensure a fair comparison between IRM and MMRM, PK data were not incorporated into the IRM analysis and only EXACT data were analyzed. Daily records were obtained by the completion of the EXACT tool using an electronic diary (3). The EXACT tool is a 14-item PRO instrument designed to capture information on the occurrence, frequency, severity, and duration of symptoms suggestive of disease exacerbation in patients with COPD. Nine out of 14 questions include five ordered categorical response options, and five questions have four categories (Table I). The total score for EXACT (EXACT-Total) ranges from 0 to 100, with higher scores indicating more severe symptoms. The E-RS:COPD consists of 11 items from the 14-item EXACT instrument with a scoring range of 0–40 (RS-Total) and captures information related to the respiratory symptoms of COPD (breathlessness, cough, sputum production, chest congestion, and chest tightness). Furthermore, three subscales assess breathlessness (RS-Breathlessness, subscale score ranges from 0 to 17), cough and sputum (RS-Cough and Sputum, subscale score ranges from 0 to 11), and chest-related symptoms (RS-Chest Symptoms, subscale score ranges from 0 to 12) (Table I). The electronic diary did not allow patients to skip individual items to avoid incomplete entries; however, missing days where patients did not provide an answer for any of the items were possible.

**Table I:** Content of the EXACT and E-RS:COPD Scales^a^ (3)

Item number	Item-level construct	Score	Symptom construct
7	Breathless today	0–4	Breathlessness
8	Breathless with activity	0–3	
9	Short of breath – personal care	0–4	
10	Short of breath – indoor activities	0–3	
11	Short of breath – outdoor activities	0–3	
2	Cough frequency	0–4	Cough and sputum
3	Mucus quantity	0–3	
4	Difficulty with mucus	0–4	
1	Congestion	0–4	Chest symptoms
5	Discomfort	0–4	
6	Tightness	0–4	
12	Tired or weak	0–4	Additional attributes
13	Sleep disturbance	0–4	
14	Scared or worried	0–3	

^a^All 14 items are administered as a daily electronic diary; the EXACT total score uses all 14 items with logit scoring transformed to a 0 to 100 interval-level scale; E-RS:COPD scoring uses only the respiratory symptom items, with subscales for breathlessness, cough and sputum, and chest symptoms. E-RS:COPD scores are based on summation to yield ordinal-level scales with a total score ranging from 0 to 40 (3)

### IRM Building

The IRM was developed by firstly determining the item characteristic functions (ICFs) (step 1), and secondly by developing the longitudinal model (step 2).

#### Item Characteristic Functions

Non-linear ICFs describing the relationship between the unobserved patient’s disease status (e.g. COPD disease severity), also known as a latent variable (ψ), and the probability for giving a certain response for an item were determined. An “independent occasion” approach (19) was used to develop the base IRT model. This approach assumes that measurement data (e.g. EXACT data) from each patient and occasion (time when data were reported) are treated independently. To do this, each measurement occasion was treated as a separate individual (different ID values at different occasions) assuming that *ψ* follows a normal distribution with fixed mean and variance *N*(0,1) at baseline and estimated mean and variance *N*(*μ*,*ω*^2^) at later occasions.

Observed data are the daily EXACT scores for an individual *i* and an item *j* defined as *y*_*ij*_. A logistic transformation was used to model each item (*j*) (Eq. 1 and Eq. 2), where *P*(*y*_*ij*_ ≥ k) is the probability of patient *i* reporting a response (*y*) at or above item score *k* (Eq. 1) and *P*(*y*_*ij*_ = *k*) is the probability of rating exactly score *k* (Eq. 2); *ψ*_*i*_ is the latent variable of patient *i*, and *a*_*j*_ and *b*_*j,k*_ are fixed effect item parameters representing discrimination and difficulty parameters for item *j*; more specifically, *b*_*j,k*_ is the difficulty parameter for the item score *k*.

1 $$ P\left({y}_{ij}\ge k\right)=\frac{e^{\left({a}_j\left({\psi}_i-{b}_{j,k}\right)\right)}}{1+{e}^{\left({a}_j\left({\psi}_i-{b}_{j,k}\right)\right)}} $$

2 $$ P\left({y}_{ij}=k\right)=P\left({y}_{ij}\ge k\right)-P\left({y}_{ij}\ge k+1\right) $$

#### Longitudinal Model

Since in the previous step, each time point was considered as a separate individual to inform ICF parameters, in this step, a data reconciliation was needed to include each individual’s time-course data and thus develop the longitudinal model with ICF parameters fixed from the previous step. Linear and non-linear functions were investigated to describe changes in individual symptoms-time course (*ψ*_*i*_). These functions included a linear (Eq. 3), power (Eq. 4), asymptotic (Eq. 5), Weibull (Eq. 6), and step function (Eq. 7). As the aim was to assess the difference between the arms, different parameters per arm (except baseline) were estimated for the model.

3 $$ {\psi}_i={\psi}_{i,t=0}+{\mathrm{slope}}_i\cdotp t $$

4 $$ {\psi}_i={\psi}_{i,t=0}+{\mathrm{slope}}_i\cdotp {t}^{\gamma } $$

5 $$ {\psi}_i={\psi}_{i,t=0}+\left({R}_{\operatorname{MAX}i}-{\psi}_{i,t=0}\right)\cdot \left(1-e\left(-\frac{\ln (2)}{T_{\mathrm{PROG}i}}\cdot t\right)\right) $$

6 $$ {\psi}_i={\psi}_{i,t=0}+{R}_{\operatorname{MAX}i}\cdotp \left(1-e\left(-{\left(\frac{\ln (2)}{T_{\mathrm{PROG}i}}\cdotp t\right)}^{\gamma}\right)\right) $$

7 $$ {\psi}_i=\left\{\begin{array}{l}{\psi}_{i,t=0}+{R}_{\operatorname{MAX}i}\\ {}{\psi}_{i,t=0}\end{array}\right.\kern2.25em {\displaystyle \begin{array}{l}\mathrm{for}\ t>{T}_{Ri}\\ {}\mathrm{for}\ t\le {T}_{Ri}\end{array}} $$

Model parameters such as slope_*i*,_ *T*_PROG*i*_ (disease progression time), *R*_MAX*i*_ (maximum response), *T*_*Ri*_ (time of response), and *ψ*_*i,t =* 0_ (baseline latent variable with a mean value fixed to 0) are subject-specific parameters with inter-individual variability (IIV) following a normal distribution with a mean of 0 and variance *ω*^2^. *T*_PROG*i*_ and *T*_*Ri*_ were assumed to be log-normally distributed with IIV modelled using an exponential function, whereas all other parameters were assumed to be normally distributed with an additive IIV model. Time is represented by *t*, and *γ* is the gamma value that governs the steepness described by the Weibull function.

A 14-item-specific longitudinal 4–5 state-minimal continuous-time Markov model (20) with a linear time-dependency on mean equilibrium time (MET) was utilized to account for the correlation between observations (16). The first IRM with Markovian properties was described by Germovsek *et al.* (16). Germovsek and colleagues used a minimal continuous-time Markov model on an individual item level where the next observation depended only on the current observation (first-order MM). IIV was included on MET using an exponential function. In the current analysis, the same minimal continuous-time Markov model was incorporated but MET was re-estimated with the available data.

### Software and Estimation Method

The software NONMEM (ICON Development Solutions, Ellicott City, Maryland) version 7.4.4 (21) was used for modeling and simulation together with an Intel FORTRAN compiler and Perl-speaks-NONMEM (PsN, http://psn.sourceforge.net) version 4.9.5 (22). R software (The R Foundation for Statistical Computing) version 3.5.2 (23) and R packages, such as Xpose4 (http: //xpose.sourceforge.net, version 4.6.1) (24, 25), Piraid (version 0.4) (26), and pROC (version 1.16.2) (27), were used for data management as well as to perform graphical analysis, produce summary statistics, and examine the NONMEM outputs.

For estimating ICFs (step 1), first-order conditional estimation method with Laplace approximation (LAPLACE) was used, whereas for the estimation of parameters in the longitudinal model (step 2), the Monte Carlo importance sampling (IMP) was used as, in contrast to step 1, most parameters included random effects.

### Model Discrimination and Internal Model Evaluation

In step 1, non-parametric ICF smooth plots were developed to assess ICF fit. An agreement between observed and simulated smooths indicates an acceptable model fit (28).

In step 2, model selection was based on parameter plausibility and the objective function value (OFV). The likelihood ratio test was used to compare nested models with a significance level of 5% for selecting a more complex model. The Akaike information criterion (AIC) was used for non-nested models.

The predictive performance of the model was assessed by using visual predictive check plots (VPCs), where the 2.5th, 50th, and 97.5th percentile of the observed data were compared to the 95%CI for the 2.5th, 50th, and 97.5th percentiles of the simulated (*N* = 500) data. VPCs were produced on the individual item score level, stratified and non-stratified by items, and on the EXACT-Total score level. In addition, a VPC for transitions was made to evaluate the Markov part of the model, as described previously (16). All VPCs were stratified by treatment arm.

### Simulations Propagating Parameter Uncertainty

#### Precision in Clinical Trial Endpoint

Precision in clinical trial endpoint was obtained by including uncertainty in IRM parameter estimates. EXACT-Total, RS-Total, and subscale scores at month 12, linked to the individual patient disease status (ψ_i_), were simulated using the final IRM parameter estimates. The derived relationship between disease status and EXACT-Total, RS-Total, and subscale scores, which was used as a basis in the simulations, is shown in Fig. S1. These stochastic (Monte Carlo) simulations included parameter uncertainty from the estimated asymptotic variance-covariance matrix of the estimates by using the $PRIOR functionality in NONMEM. Specifically, NWPRI subroutine was used where prior fixed and random effects are assumed to be normally and inverse-Wishart distributed, respectively. Degrees of freedom for the inverse-Wishart distribution were calculated based on standard error (SE) of estimates (29). As illustrated in Fig. S2, EXACT-Total, RS-Total, and subscale scores were simulated (*N* = 2000) for each treatment arm, using a large population (*N*_subj_ = 5000 per arm), to obtain an expected difference distribution in observed score between arms (average total score in drug arm minus average total score in placebo arm) that included parameter uncertainty. The median, 2.5th, and 97.5th percentiles of the resulting 2000 arm-differences in mean score were used to represent mean drug effect (95%CI). These IRM-derived values were compared with those (published values) obtained at month 12 using the MMRM analysis (18).

#### Sample Size and Probabilities of Correct and Incorrect Stop/Go Decision

Sample size (*N*) comparison of IRM relative to MMRM CI values was calculated considering the precision obtained from the MMRM (95%CI length - CI_MMRM_) and the IRM (95%CI length - CI_IRM_) as the desired margin of error (Eq. 8).

8 $$ N={\left(\frac{{\mathrm{CI}}_{\mathrm{MMRM}}}{{\mathrm{CI}}_{\mathrm{IRM}}}\right)}^2 $$

The relative merits of the two methods were further explored in simulations. Considering uncertainty obtained from IRM and MMRM, the probabilities of giving a correct/incorrect go decision (*P*(Correct go)/*P*(Incorrect go)) as well as probabilities of correct/incorrect stop decision (*P*(Correct stop)/*P*(Incorrect stop)), yielding a total probability of go (*P*(Go)) or stop (*P*(Stop)) decision, was calculated conditionally on a true treatment effect Δ_T_ (total score in drug arm minus total score in placebo arm) as described previously (30, 31). This true treatment effect followed a mixture distribution assuming that 80% of the mixture is having a point mass at zero, and the remaining 20% of the mixture follows a normal distribution centered to a target value (TV) with a standard distribution of 1 (Fig. S3). This TV was chosen based on a minimum clinically important significant difference value of −2 for both EXACT and E-RS:COPD, −1 for RS-Breathlessness, and − 0.7 for both RS-Cough and Sputum and RS-Chest Symptoms (6). A *P*(Correct go) requires that both the Δ_T_ and the treatment effect simulated from the IRM (Δ_IRM_) or MMRM (Δ_MMRM_) approaches is equal or lower than the TV, whereas a *P*(Correct stop) decision was defined as both the Δ_T_ and Δ_IRM or MMRM_ being higher than the TV (Fig. S3). Probabilities were calculated based on 10000 simulated independent samples following a normal distribution with a mean of Δ_T_ and a standard deviation (SD) of the treatment difference (drug minus placebo) for each approach (IRM and MMRM) as illustrated in Fig. S3. Positive predictive values (PPV) and negative predictive values (NPV) were also calculated as shown in Eq. 9 and Eq. 10 (30).

9 $$ \mathrm{PPV}=\frac{P\left(\mathrm{Correct}\ \mathrm{go}\right)}{P\left(\mathrm{Go}\right)} $$

10 $$ \mathrm{NPV}=\frac{P\left(\mathrm{Correct}\ \mathrm{stop}\right)}{P\left(\mathrm{Stop}\right)} $$

#### Power Function and Sensitivity/Specificity of the IRM and MMRM Analyses

A power function that gives *P*(Go) and *P*(Stop) decision for various values of the efficacy endpoint and a receiver operating characteristic curve (ROC) to assess the sensitivity and specificity of each approach were developed. Equations 11 and 12 were used to calculate these *P*(Go) and *P*(Stop), respectively. The SD of the treatment arms difference (σ_Δ_) for the IRM was obtained from simulations (illustrated in Fig. S2), whereas for the MMRM, published values (EXACT: 2.70 and E-RS:COPD: 1.74) were considered. TV is the target value described in the paragraph above.

11 $$ P\left(\mathrm{Go}\right)=1-\Phi \left(\frac{\mathrm{TV}-\Delta  }{\upsigma_{\Delta  }}\right) $$

12 $$ P\left(\mathrm{Stop}\right)=1-\mathrm{P}\left(\mathrm{Go}\right) $$

For the ROC curve development, the distribution of the EXACT-Total, RS-Total, and subscale scores per treatment arm obtained from the IRM and MMRM analysis was considered. For the IRM, precision was obtained from the distribution of 2000 simulated EXACT-Total, RS-Total, and subscale scores for drug and placebo arm (Fig. S2), and for the MMRM, a reported mean and SE for EXACT-Total, RS-Total, and subscale scores were used (18) (Table S3).

## RESULTS

### Clinical Studies and Patients

Data were available from 93 patients (mean [SD] age of 60.5 years (7.31), 73.1% smokers at study initiation) who received either oral danirixin 75 mg twice daily (*n* = 45) or placebo (*n* = 48) for 52 weeks (Fig. S4). Seventy-five patients (81%) provided data at least up to week 52 with a median (range) missing days of 9 (0–134), whereas 18 patients (19%) stopped filling out the questionnaire after 131 (6–345) days with 1 (0–46) missing days. Baseline characteristics are shown in Table II.

**Table II:** Patient Characteristics at Baseline. Values as Presented as Mean (SD) or Number (%)

Baseline characteristics	Danirixin 75 mg twice daily (*n* = 45)	Placebo (*n* = 48)
Age (years)	62.4 (6.91)	58.8 (7.32)
FVC (L)	3.28 (1.01)	3.39 (0.99)
FEV_1_ (L)	1.77 (0.64)	1.77 (0.52)
Male (*n*)	22 (49%)	23 (48%)
Smoker (*n*)	34 (76%)	34 (71%)
COPD GOLD disease status	Mild: 9 (20%)	Mild: 10 (21%)
	Moderate 36 (80%)	Moderate: 38 (79%)
EXACT-Total	35.6 (9.78)	36.1 (10.6)
RS-Total	11.2 (5.81)	11.4 (6.59)

*FVC*, forced vital capacity; *FEV*_*1*_, forced expiratory volume in one second; *GOLD*, global initiative for chronic obstructive lung disease. EXACT-Total score based on logit transformed data (ranged from 0 to 100); RS-Total score based on summation to yield ordinal-level scales (ranged from 0 to 40)

### IRM and Simulations

**Fig. 1:**
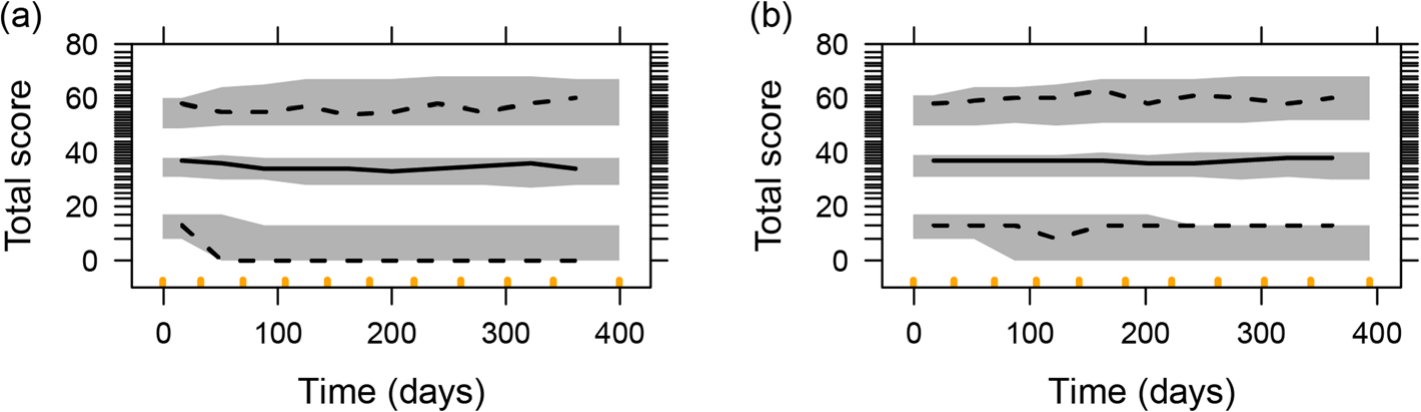
Visual predictive check (500 simulations) for the EXACT-Total score (logit score-transformed 0–100) in the treatment (**a**) and placebo (**b**) arms. Lines are the 2.5th, 50th, and 97.5th percentile of the observed data, and grey areas are the corresponding 95% confidence interval from model simulations

**Fig. 2:**
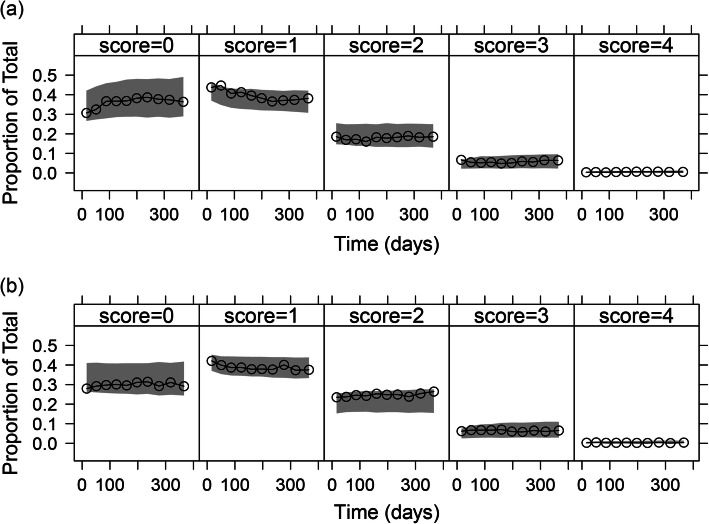
Visual predictive check for item scores of all 14 items in the treatment (**a**) and placebo (**b**) arms. Lines correspond to different proportion of observations and grey areas are the 95% confidence intervals (500 simulations)

ICF parameters were estimated with good precision (Table S1). Item characteristic curves showing the relationship between disease status and probability of giving a certain score for all items are shown in Fig. S5. A step function best described the COPD symptoms-time course in both danirixin and placebo arms, and different parameters per arm were estimated with a median (range) relative standard error (RSE) of 0.15 (0.06–1.09) (Table S2). This model showed a satisfactory fit to the total score data, as seen with agreement between observed and simulated percentiles in a VPC (Fig. 1). VPCs on the item score level of all 14 items and stratified by individual items are shown in Fig. 2 and Fig. S6a–f, respectively. Typical (SE) *R*_MAX_ and *T*_*R*_ were − 0.16 (0.18) and 54.8 days (15.6) (danirixin) and 0.18 (0.15) and 51.1 days (18.9) (placebo), respectively. The typical MET (SE) was 3.09 days (0.41) at the end of the study (i.e. day 365), and 1.23 days (0.08) at the beginning of the study (i.e. day 0) (Table S2). Transitions were well described by the model as is shown in Fig. S7. The IRM model included 70 item-related parameters (five fixed), and 14 longitudinal-related parameters (one fixed) compared to 117 parameters in the MMRM. Note that the estimation of the item-related parameters was not performed using allocation information.

**Fig. 3:**
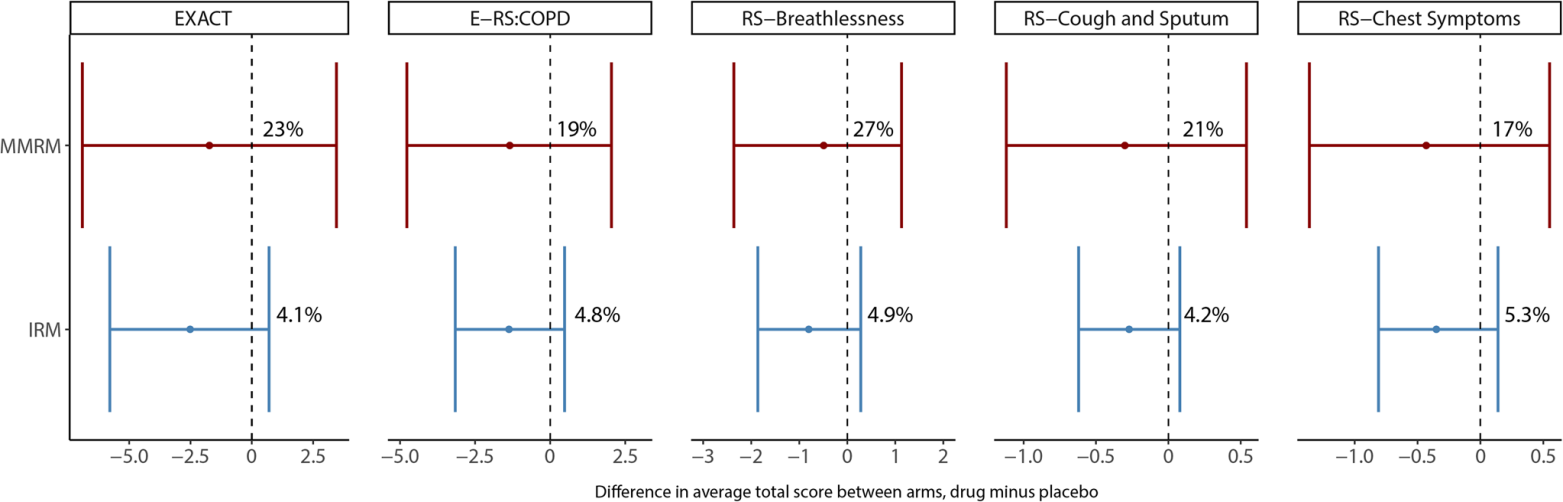
Mean (95%CI) difference in average EXACT-Total, RS-Total, and subscale scores between arms using a MMRM and IRM analysis. For the MMRM analysis, the percentages are the proportion of mean treatment differences greater than 0 derived from the **Z**-score (using the standard deviation for 95% CI equal-tailed). For the IRM, the percentages correspond to the number of simulated arm-differences with a mean greater than zero

The IRM considerably improved the precision of the drug effect compared to the MMRM (Fig. 3 and Table S3) in all scales explored in this one case study. For instance, with the E-RS:COPD scale, the mean (95%CI) difference in average total score between arms using the IRM was −1.37 (−3.16, 0.48) compared to −1.35 (−4.77, 2.04) for the MMRM analysis at month 12, meaning the uncertainty (CI width) decreased from 6.81 to 3.64. Furthermore, a sample size (obtained using Eq. 8) of 2.5 and 3.5 times larger would be required in the MMRM analysis to achieve the precision obtained with the IRM analysis using EXACT and E-RS:COPD, respectively. As shown in Fig. 3, with MMRM, a higher percentage of mean treatment differences is above 0 compared to IRM (including all scales). This means that with MMRM, a higher percent of the time the drug effect may not be confirmed, although it is important to highlight that none of the methods resulted in a significant drug effect.

The *P*(Correct stop), *P*(Incorrect stop), *P*(Correct go), *P*(Incorrect go), *P*(Stop), and *P*(Go) for both approaches (IRM and MMRM) considering EXACT and E-RS:COPD scales are shown in Table III. The IRM analysis gave a higher *P*(Correct stop) than the MMRM analysis, and the *P*(Incorrect go) was higher in the MMRM approach compared to that in IRM. No difference was seen in the probability of giving a *P*(Incorrect stop) and *P*(Correct go) between approaches using both scales (Table III).

**Table III:** Probabilities of Correct or Incorrect Positive (Go) and Negative Decisions (Stop), and Positive/Negative Predictive Values (PPV/NPV) for a Target Value (TV) of −2 (EXACT and E-RS:COPD)

	EXACT				E-RS:COPD			
	IRM		MMRM		IRM		MMRM	
Decision	Stop	Go	Stop	Go	Stop	Go	Stop	Go
Δ_T_ > TV	**0.78**	0.12	**0.68**	0.22	**0.87**	0.04	**0.77**	0.13
Δ_T_ ≤ TV	0.03	**0.07**	0.04	**0.06**	0.02	**0.07**	0.03	**0.07**
Total	0.81	0.19	0.72	0.28	0.89	0.11	0.80	0.20
PPV	0.34		0.22		0.67		0.34	
NPV	0.96		0.95		0.97		0.96	

*Δ*_*T*_, true drug effect; *PPV*, positive predictive value; *NPV*, negative predictive value. PPV and NPV values were calculated including all available significant digits. Values in bold represent the *P*(Correct stop) and *P*(Correct go) decisions

The two approaches showed a similar performance to estimate NPV, but differences were seen for PPV with the IRM showing a higher probability of making a correct decision when there is a true drug effect (34% [EXACT] and 67% [E-RS:COPD]) compared to MMRM (22% [EXACT] and 34% [E-RS:COPD]) (Table III). The *P*(Correct go), *P*(Incorrect go), *P*(Correct stop), *P*(Incorrect stop), *P*(Stop), *P*(Go), PPV, and NPV results obtained for E-RS:COPD subscales are shown in Table S4.

**Fig. 4:**
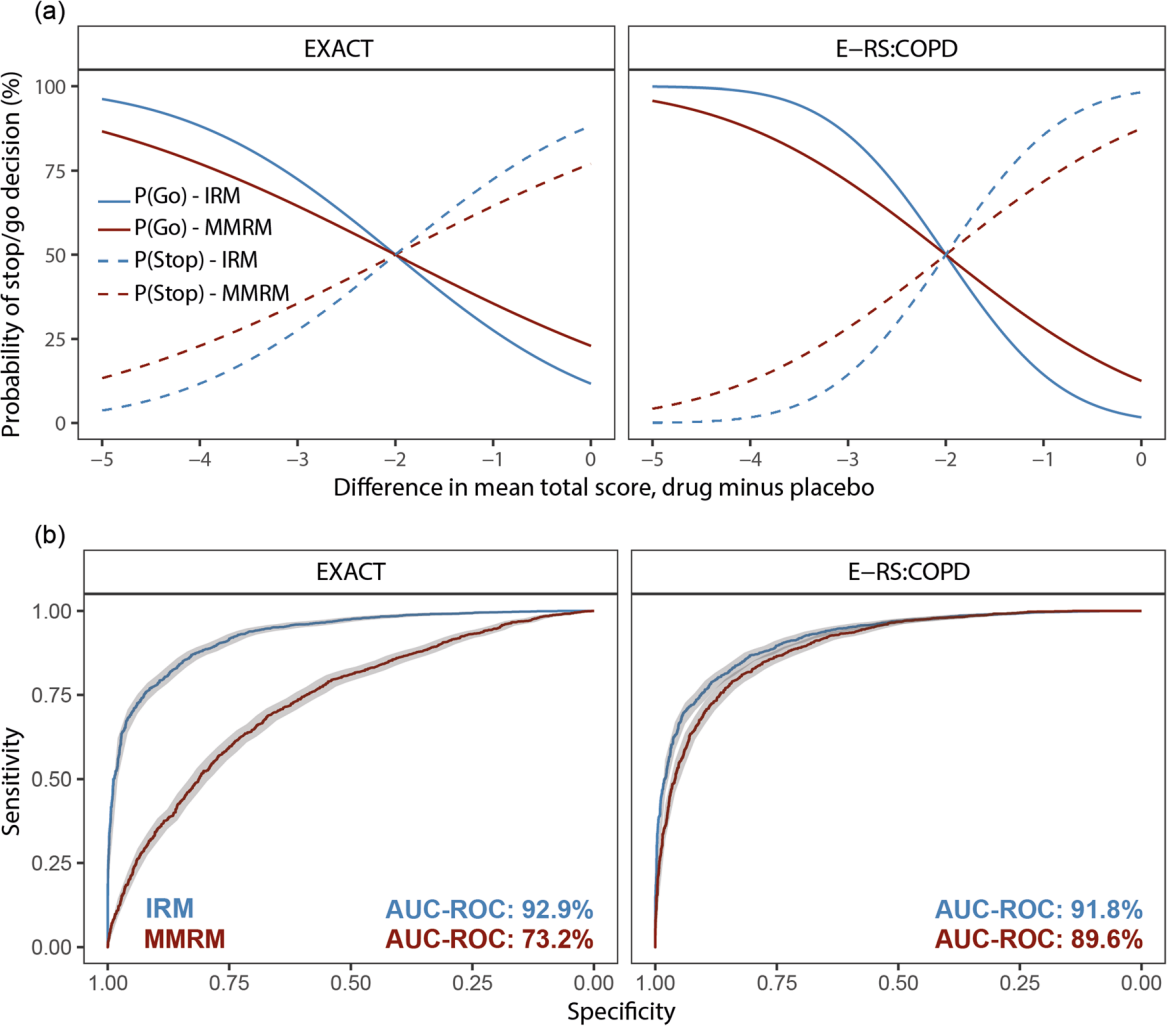
Probabilities of stop and go decision over a range of drug effect values (**a**) and ROC curves (**b**) for the IRM and MMRM analysis using EXACT and E-RS:COPD scales. AUC-ROC corresponds to the area under the ROC curve, and the grey areas correspond to the 95%CI of the ROC curve

A higher probability to detect a drug effect and, for example, make a go decision was observed with IRM (Fig. 4a). A power of 80% or greater was seen with a drug effect (difference in total score between arms, drug minus placebo) of at least −3.38 (EXACT) and − 2.78 (E-RS:COPD) with IRM compared to −4.22 (EXACT) and − 3.43 (E-RS:COPD) with MMRM. The IRM approach also showed a better precision around the mean EXACT-Total, RS-Total, and subscale scores for both drug and placebo arms (Table S3), as well as better performance at controlling true and false positive rates with an area under the ROC curve (AUC-ROC) [95%CI] of 92.9% [92.1–93.6] (EXACT) and 91.8% [91.0–92.6] (E-RS:COPD) compared to 73.2% [71.7–74.8] (EXACT) and 89.6% [88.6–90.5] (E-RS:COPD) (Fig. 4b). ROC curves for E-RS:COPD subscales are shown in Fig. S8.

These results show that a much smaller sample size would have been required with the IRM to arrive to the same conclusion as with MMRM (e.g. no significant drug effect). Furthermore, due to its higher precision, a higher probability of making a correct decision, hence greater confidence, can be achieved with IRM compared to MMRM.

## DISCUSSION

In this study, a NLME analysis based on item-level data (IRM) has been proposed as an alternative to MMRM for efficacy evaluation. Based on stochastic simulations, the IRM improved the precision of the estimated drug effect considerably compared to published MMRM analysis results. Consequently, analysis with an IRM may help to take more precise and unbiased decisions as well as significantly reduce study sample size to show drug effect. For example, a 2.5-fold (EXACT) or 3.5-fold (E-RS:COPD) smaller study size with IRM compared to MMRM analysis appeared necessary. The benefit of using a NLME to improve power in clinical trials has been shown previously, where a 4.3-fold difference in total size study between a NLME and *t* test analysis was shown. While Karlsson *et al.* (32) calculated sample size based on the hypothesis testing principle of the likelihood ratio test in NLME, the sample size in this study was obtained comparing the same primary endpoint (arm-difference in total score) for the two analyses (IRM and MMRM) using observed clinical trial data. Moreover, IRM analysis has displayed a consistently higher power to detect a drug effect than other methods such as least-square means analysis, with 71% fewer subjects to achieve 80% power (14). The benefit of using IRM for decision-making in drug development analysis has already been explored, demonstrating how IRM may have an impact on inclusion criteria decisions. For example, IRM can provide answers to questions related to patient disease status linked to probability to detect a drug effect (33).

Simulations using a NLME model can be useful not only for power calculation but also for predicting outcome of future trials such as probability of success or failure as shown in this study. Furthermore, the longitudinal nature of the NLME allows the prediction of a stop/go decision at different time points during the clinical trial, which can be useful in early clinical development. In this study, *P*(Go) and *P*(Stop) decisions were simulated where the IRM consistently showed a better performance than MMRM at handling type I and II errors (Table III). These results are dependent on both the assumptions considered in this study and the chosen TV. A mixture distribution for the true treatment effect used in this study assumes that one out of five compounds is effective. This reflects the effect size seen for all compounds in the pharmaceutical industry in the recent decades (34), where 20% is often the percentage of a new molecular entity to reach the registration phase among those entering phase II (31).

The better precision obtained with IRM using both scales (EXACT and E-RS:COPD) makes this approach more informative with a higher *P*(Go) or *P*(Stop) decision when the drug effect either goes beyond the TV or closer to zero, respectively (Fig. 4a). For example, when the drug effect is 2.5 times higher than the TV (around −5), the *P*(Go) is 96% (IRM-EXACT) compared to 87% (MMRM-EXACT) and 100% (IRM-E-RS:COPD) compared to 96% (MMRM-E-RS:COPD). The same trend can be observed when comparing scales (EXACT vs. E-RS:COPD). Here, E-RS:COPD showed a better precision around the efficacy endpoint than EXACT (Fig. 3) as well as lower incidence of type I and II errors (Table III). Using EXACT, the probability of having a go decision of 100% is not reached even though the drug effect is 2.5 times bigger than the TV (Fig. 4a). This may suggest that E-RS:COPD might be more informative than EXACT scale in this particular population that includes patients with mild or moderate COPD severity (Table II). Although study design and endpoint selection is an important factor, it could also be hypothesised that the better performance of E-RS:COPD compared to EXACT is due to the fact that the latter was designed to measure changes in symptoms suggestive of an exacerbation, which are usually characterized by an acute and short expression of symptoms in the patient’s COPD, while E-RS:COPD excludes the items related to more acute exacerbation events, although still measures ongoing respiratory symptoms. Furthermore, another explanation could be obtained by observing the discrimination parameter values for those items that are not included in E-RS:COPD (items 12, 13, and 14). These values are 1.24, 0.56, and 1.05, respectively (Table S1). According to Baker (35), a discrimination value between 0.65 and 1.34 can be defined as moderate, whereas between 0.35 and 0.64, it can be defined as low. This means that these three items can only provide low/moderate differentiation between patients in this particular population, making the EXACT scale less discriminatory than E-RS:COPD.

Additionally, the ROC curves presented in this study not only show a better sensitivity and specificity for IRM but also show that IRM gives consistent results across scales (1% difference in AUC-ROC between EXACT and E-RS:COPD). While the MMRM appears more sensitive to the scale of choice, with a difference of 16% in AUC-ROC between EXACT and E-RS:COPD, this must be interpreted cautiously since the ROC curve is highly dependent on the mean difference between arms (which may vary depending on random processes). Comparing the two scales, the substantial higher AUC-ROC for IRM over MMRM with the EXACT scale may be explained by the combination of both a higher mean difference in total score (drug minus placebo: −2.4 vs. −1.7) and an increased precision with IRM (Table S3). Conversely, for the E-RS:COPD scale, a higher mean difference in total score is observed with MMRM (−2.0 vs. −1.3); however, the greater precision with the IRM still results in a slightly higher AUC-ROC (Fig. 4b).

To the best of our knowledge, only one study has compared a NLME model to MMRM but using simulated data set. The NLME analysis was shown to be more powerful than MMRM in some (albeit not all) scenarios (17), which may be due to study design and/or model misspecification. MMRM has been widely used to analyze longitudinal data, and it has shown to be less biased, particularly for handling missing data, than other methods such as last observation carried forward (11). In the case of a NLME-IRM analysis, item responses that are missing completely at random can be ignored without the need for imputation, whereas missing data on the longitudinal level can be handled in the same way as is done with any other NLME model, for example, by single imputation (substitution by median, mean, or mode value of population) or imputing expected value based on other variable. In this study, the nature of the electronic diary did not allow partial/incomplete missing data, although missing days were possible when patient did not provide an answer for any of the items. It was observed, in this study, that total score data were not influenced by the drop-outs; therefore, a drop-out model was not deemed necessary. Although MMRM is considered the gold standard approach, it may produce biased results when the correlation structure is misspecified (36) or when non-ignorable missing (missing not at random) data patterns are presented. As such, sensitivity analysis may be required to assess the impact that missing not at random data may have on the estimated results (37).

While the present results are encouraging and are based on a real clinical dataset, this analysis represents one case study. To make stronger conclusions about the potential to replace MMRM with IRM for the analysis of end-of-treatment item-based data, future research work could focus on investigating the following: (i) the accuracy of the SE’s obtained with a model-based analysis. Clinical trial simulations could be contemplated; (ii) model uncertainty and its impact on the precision around the efficacy endpoint. It has been already discussed that model averaging has advantages to mitigate downward bias in model uncertainty in a NLME model–based analysis (38, 39), and (iii) the accuracy of using the SE from the asymptotic variance-covariance matrix in NONMEM. The variance-covariance matrix, bootstrap, or sampling importance resampling (SIR) (40) may lead to different uncertainty estimates, and it is difficult to know which method is the most adequate in a given case. The authors acknowledge that assumptions are made about the uncertainty distribution with the variance-covariance matrix; however, the comparison between methods and the impact of the different SE applied in the simulations was not in the scope of this analysis.

The use of a NLME model–based approach in drug development and the positive impact of using model simulations in decision-making process have been already discussed (41–43). This one case study not only shows the advantage of using a NLME model over a standard approach used today in drug development (MMRM) for the same endpoint but also exemplifies how it may help in predicting future trial outcomes (*P*(Go) and *P*(Stop) decisions). Specifically, the IRM in this study provided a considerably more informed basis for assessing the drug effect and it may improve decision-making in phase II of drug development; however, further analysis should be performed to confirm these findings.

## Supplementary Information

ESM 1 (DOCX 11838 kb)
