# Supplementary material for: Improved Decision-Making Confidence Using Item-Based Pharmacometric Model: Illustration with a Phase II Placebo-Controlled Trial
Source: AAPS J. 2021 Jun 2;23(4):79. doi: 10.1208/s12248-021-00600-1 (PMC8172506; doi:10.1208/s12248-021-00600-1)

**Improved decision-making confidence using item-based pharmacometric model: illustration with a Phase II placebo-controlled trial**

Carolina Llanos-Paez^1^, Claire Ambery^2^, Shuying Yang^2^, Maggie Tabberer^3^, Misba Beerahee^2^, Elodie L. Plan^1^, Mats O. Karlsson^1^

1. Department of Pharmacy, Uppsala University, Uppsala, Sweden

2. Clinical Pharmacology Modelling and Simulation, GlaxoSmithKline plc., London, UK

3. Patient Centred Outcomes: Value Evidence and Outcomes, GlaxoSmithKline plc., Brentford, Middlesex, UK

***Corresponding author**: Prof. Mats O. Karlsson, Department of Pharmacy, Uppsala University, Box 580, 751 23 Uppsala, Sweden. Email: [mats.karlsson@farmaci.uu.se](mailto:mats.karlsson@farmaci.uu.se)

**Supplementary material**

## Tables

## Table S1. Item characteristic parameter estimates (Step 1)

| **Parameter** | **Mean (SE)** | **Parameter** | **Mean (SE)** |
| --- | --- | --- | --- |
| DIS_i1 | 1.05 (0.11) | DIS_i8 | 1.91 (0.20) |
| DIF1_i1 | -1.54 (0.22) | DIF1_i8 | -1.24 (0.19) |
| DIF2_i1 | 2.75 (0.28) | DIF2_i8 | 2.14 (0.22) |
| DIF2_i1 | 2.84 (0.29) | DIF2_i8 | 1.52 (0.16) |
| DIF4_i1 | 3.08 (0.34) | DIF4_i8 | 0 FIX |
| DIS_i2 | 0.868 (0.09) | DIS_i9 | 2.84 (0.29) |
| DIF1_i2 | -3.00 (0.34) | DIF1_i9 | 0.14 (0.14) |
| DIF2_i2 | 3.14 (0.32) | DIF2_i9 | 1.10 (0.11) |
| DIF3_i2 | 2.76 (0.28) | DIF3_i9 | 1.44 (0.15) |
| DIF4_i2 | 4.47 (0.47) | DIF4_i9 | 2.19 (0.23) |
| DIS_i3 | 0.505 (0.05) | DIS_i10 | 3.25 (0.34) |
| DIF1_i3 | -3.11 (0.36) | DIF1_i10 | -0.48 (0.12) |
| DIF2_i3 | 9.35 (0.97) | DIF2_i10 | 1.43 (0.15) |
| DIF3_i3 | 7.36 (0.82) | DIF3_i10 | 1.36 (0.14) |
| DIF4_i3 | 0 FIX | DIF4_i10 | 0 FIX |
| DIS_i4 | 1.07 (0.11) | DIS_i11 | 2.30 (0.24) |
| DIF1_i4 | -0.62 (0.16) | DIF1_i11 | -0.82 (0.17) |
| DIF2_i4 | 1.83 (0.19) | DIF2_i11 | 1.45 (0.15) |
| DIF3_i4 | 1.91 (0.20) | DIF3_i11 | 1.38 (0.14) |
| DIF4_i4 | 1.97 (0.21) | DIF4_i11 | 0 FIX |
| DIS_i5 | 1.68 (0.17) | DIS_i12 | 1.24 (0.13) |
| DIF1_i5 | 0.02 (0.14) | DIF1_i12 | -0.93 (0.17) |
| DIF2_i5 | 1.62 (0.17) | DIF2_i12 | 2.28 (0.23) |
| DIF3_i5 | 2.26 (0.23) | DIF3_i12 | 1.68 (0.17) |
| DIF4_i5 | 2.53 (0.30) | DIF4_i12 | 1.58 (0.17) |
| DIS_i6 | 1.78 (0.18) | DIS_i13 | 0.56 (0.06) |
| DIF1_i6 | 0.07 (0.14) | DIF1_i13 | -0.83 (0.17) |
| DIF2_i6 | 1.53 (0.16) | DIF2_i13 | 3.68 (0.38) |
| DIF3_i6 | 2.01 (0.21) | DIF3_i13 | 3.79 (0.40) |
| DIF4_i6 | 2.35 (0.27) | DIF4_i13 | 4.95 (0.55) |
| DIS_i7 | 3.73 (0.39) | DIS_i14 | 1.05 (0.11) |
| DIF1_i7 | -1.04 (0.18) | DIF1_i14 | 0.15 (0.14) |
| DIF2_i7 | 1.67 (0.17) | DIF2_i14 | 1.88 (0.19) |
| DIF3_i7 | 1.76 (0.18) | DIF3_i14 | 2.41 (0.25) |
| DIF4_i7 | 1.59 (0.17) | DIF4_i14 | 0 FIX |
| Ψ at time = 0 (baseline) | 0 FIX | IIV on Ψ at time = 0 (baseline) | 1 FIX |
| Ψ at time ≠ 0 | 0.12 (0.14) | IIV on Ψ at time ≠ 0 | 2. 32 (0.48) |

DIS: discrimination parameter; DIF: difficulty parameter; IIV: interindividual variability; Ψ: latent variable.

|  |  | **ω^2^** |  | | **ω^2^** |  | **ω^2^** |  | **ω^2^** |  | **ω^2^** |  | **ω^2^** |  |
| --- | --- | --- | --- | --- | --- | --- | --- | --- | --- | --- | --- | --- | --- | --- |
| **Model** | **OFV** | **Baseline** | **Slope** | | **Slope** | **T_PROG_ or T_R_** | **T_PROG_ or T_R_** | **R_MAX_** | **R_MAX_** | **MET_end** | **MET_end** | **MET_start** | **MET_start** | **γ** |
|  |  | **(RSE)** | **(RSE)** | | **(RSE)** | **(RSE)** | **(RSE)** | **(RSE)** | **(RSE)** | **(RSE)** | **(RSE)** | **(RSE)** | **(RSE)** | **(RSE)** |
| Linear | 463,945 | 1.53 (18.5) | 0.02 (38.9) | 2.49 (35.4) | | - | - | - | - | 3.12 (0.50) | 1.59 (2.74) | 1.26 (0.49) | 0.36 (9.05) | - |
| Power | 463,765 | 1.48 (0.11) | 0.06 (2.66) | 2.37 (0.13) | | - | - | - | - | 3.10 (0.13) | 1.67 (0.10) | 1.25 (0.06) | 0.36 (0.12) | 0.56 (0.31) |
| Asymptotic | 462,622 | 1.59 (0.12) | - | - | | 109.5 (0.35) | 6.97 (0.14) | 0.65 (0.58) | 8.81 (0.20) | 3.16 (0.13) | 1.57 (0.10) | 1.24 (0.06) | 0.36 (0.09) | - |
|  | (AIC = 462,640) |  |  |  |  |  |  |  |  |  |  |  |  |  |
| Weibull | 461,088 | 1.48 (0.12) | - | - | | 43.8 (0.19) | 2.11 (0.13) | 0.02 (5.68) | 1.06 (0.13) | 3.16 (0.13) | 1.60 (0.12) | 1.24 (0.06) | 0.35 (0.10) | 1e+06 (0.0003) |
| Step function | 460,919 | 1.53 (0.11) | - | - | | 62.1 (0.18) | 1.63 (0.15) | -0.01 (10.8) | 1.02 (0.13) | 3.18 (0.12) | 1.57 (0.14) | 1.23 (0.06) | 0.35 (0.12) | - |
|  | (AIC = 460,937) |  |  |  |  |  |  |  |  |  |  |  |  |  |
| Step function- drug arm | 460,895 | 1,54 (0.11) | - | - | | 54.8 (0.29) | 1.71 (0.23) | -0.16 (1.09) | 1.35 (0.15) | 3.09 (0.13) | 1.68 (0.11) | 1.23 (0.06) | 0.36 (0.09) | - |
| Step function- placebo arm |  |  |  |  |  | 51.1 (0.38) | 4.02 (0.16) | 0.18 (0.85) | 1.03 (0.08) |  |  |  |  |  |

## Table S2. Comparison between longitudinal models (Step 2).

AIC: Akaike Information Criterion (AIC  =  OFV  +  2 ⋅ number of population model parameters); OFV: Objective Function Value; MET_end: mean equilibrium time at the end of the study (days); MET_start: mean equilibrium time at the start of the study (days); R_MAX_: maximum response; RSE: relative standard error (the RSEs for omega are reported on the approximate standard deviation scale (SE/variance estimate)/2); T_PROG_: disease progression time (days), for the step function this parameter is time of maximum response (T_R_); γ: gamma parameter

## Table S3. Published results from the MMRM analysis (16) (identified with an asterisk) and results obtained from the IRM analysis for different scales/subscales.

| **Scale/subscale** | **MMRM** | **IRM** | **MMRM** | **IRM** |
| --- | --- | --- | --- | --- |
|  | **Difference in total score between arms (drug minus placebo). Mean (95% CI) [SD]** | | **Total score. Mean [SE]** | |
| EXACT | -1.73 (-6.91, 3.45)* | -2.51 (-5.79, 0.70) | Drug: 35.0 [1.91]* | Drug: 33.7 [1.26] |
|  | [2.70]* | [1.68] | Placebo: 36.7 [1.85]* | Placebo: 36.1 [1.11] |
| E-RS:COPD | -1.35 (-4.77, 2.04)* | -1.37 (-3.16, 0.48) | Drug: 10.5 [1.17]* | Drug: 10.8 [0.72] |
|  | [1.74]* | [0.94] | Placebo: 12.5 [1.13]* | Placebo: 12.1 [0.64] |
| RS-Breathlessness | -0.49 (-2.36, 1.13)* | -0.80 (-1.86, 0.28) | Drug: 4.90 [0.69]* | Drug: 5.49 [0.42] |
|  | [0.90]* | [0.55] | Placebo: 5.90 [0.62]* | Placebo: 6.28 [0.38] |
| RS-Cough and Sputum | -0.30 (-1.12, 0.54)* | -0.27 (-0.62, 0.08) | Drug: 3.20 [0.30]* | Drug: 3.32 [0.14] |
|  | [0.42]* | [0.18] | Placebo: 3.60 [0.37]* | Placebo: 3.59 [0.12] |
| RS-Chest Symptoms | -0.43 (-1.36, 0.55)* | -0.35 (-0.81, 0.14) | Drug: 2.40 [0.28]* | Drug: 2.49 [0.20] |
|  | [0.50]* | [0.25] | Placebo: 3.00 [0.31]* | Placebo: 2.84 [0.17] |

## Table S4. Probabilities of correct or incorrect positive (Go) and negative decisions (Stop), and positive/negative predictive values (PPV/NPV) for E-RS:COPD subscales

|  | **RS-Breathlessness** | | | | **RS-Cough and Sputum** | | | | **RS-Chest Symptoms** | | | |
| --- | --- | --- | --- | --- | --- | --- | --- | --- | --- | --- | --- | --- |
|  | **IRM** | | **MMRM** | | **IRM** | | **MMRM** | | **IRM** | | **MMRM** | |
| **Decision** | **Stop** | **Go** | **Stop** | **Go** | **Stop** | **Go** | **Stop** | **Go** | **Stop** | **Go** | **Stop** | **Go** |
| **Δ_T_ > TV** | 0.80 | 0.04 | 0.72 | 0.11 | 0.82 | 0.002 | 0.78 | 0.04 | 0.82 | 0.01 | 0.76 | 0.07 |
| **Δ_T_ ≤ TV** | 0.01 | 0.15 | 0.02 | 0.14 | 0.002 | 0.17 | 0.01 | 0.17 | 0.004 | 0.17 | 0.01 | 0.16 |
| **Total** | 0.81 | 0.19 | 0.74 | 0.25 | 0.82 | 0.172 | 0.79 | 0.21 | 0.82 | 0.18 | 0.77 | 0.23 |
| **PPV** | 0.79 | | 0.56 | | 0.99 | | 0.81 | | 0.94 | | 0.70 | |
| **NPV** | 0.99 | | 0.97 | | 1.00 | | 0.99 | | 1.00 | | 0.99 | |

TV: target value of -1 (RS-Breathlessness) and -0.7 (RS-Cough & Sputum and RS-Chest Symptoms), Δ_T_: true drug effect; PPV: positive predictive value; NPV: negative predictive value; PPV and NPV values were calculated including all available significant digits.

## Figures

## Fig. S1. Relationship between patient’s disease status and total scores using the EXACT (blue) and E-RS:COPD (maroon) scales as well as the RS-Breathlessness (green), RS-Cough and sputum (purple) and RS-Chest symptoms (light blue) subscales. This relationship was obtained by simulating a wide range of values of the latent variable and related them with the observed total score. For the simulations (N=10,000) the item parameters showed in Table S1 were used.


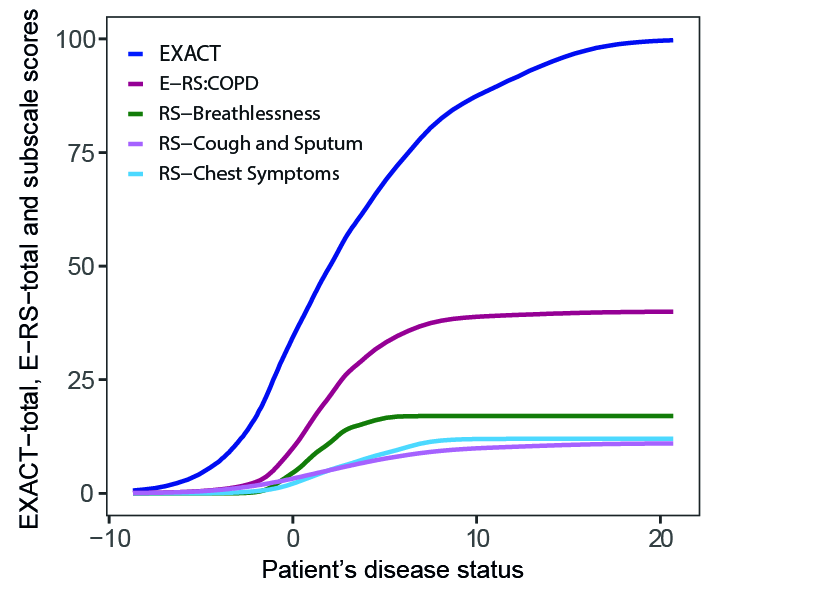


## Fig. S2. IRM simulated endpoint (difference in mean EXACT-Total/RS-Total/subscale scores between arms at month 12 – drug minus placebo). EXACT-Total, RS-Total and subscale scores were simulated (2,000 times) for each arm (drug and placebo). The 50th (2.5th, 97.5th) percentiles of the resulting 2,000 arm-differences in mean score were used to represent mean drug effect (95%CI).


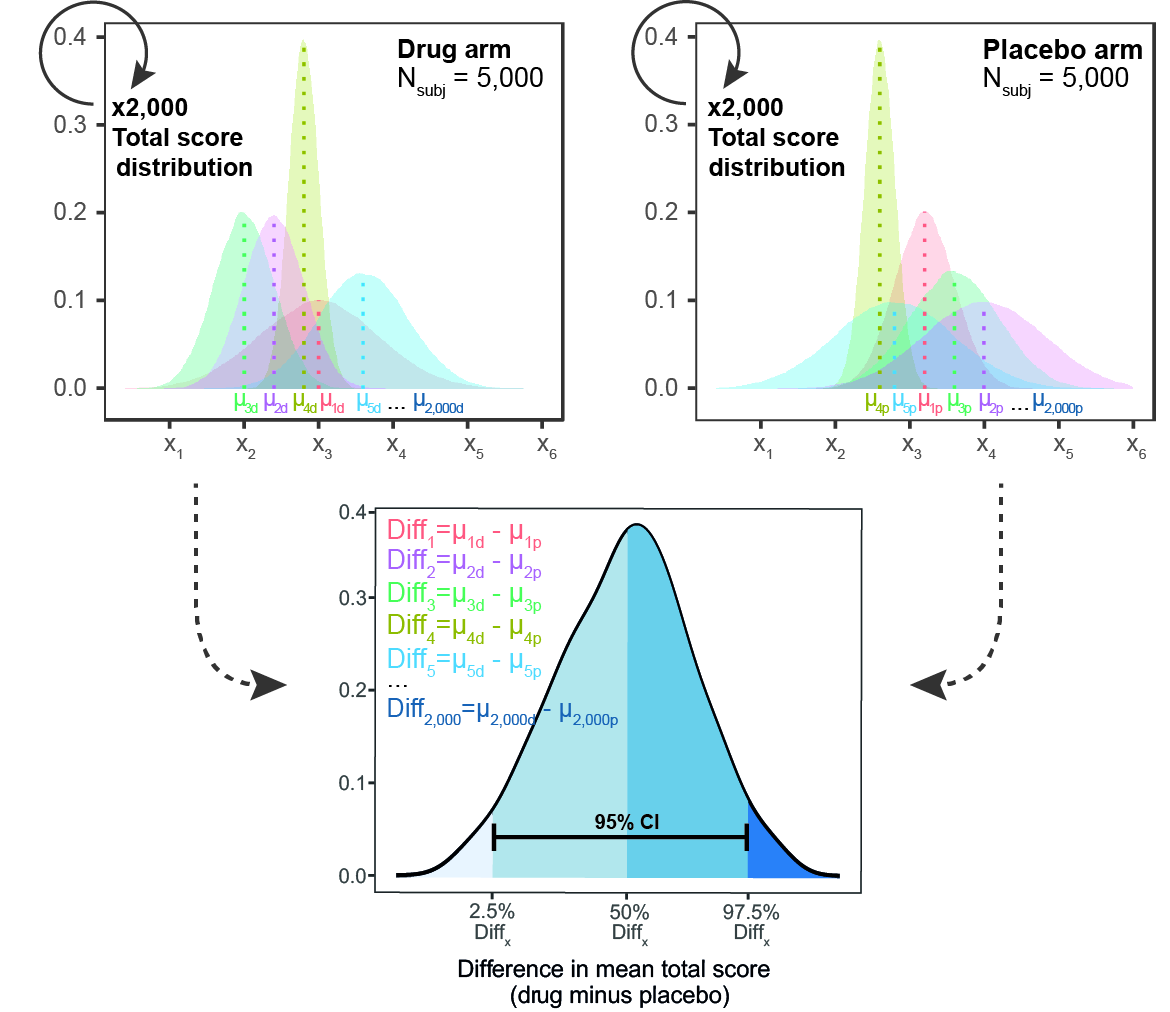


## Fig. S3. Simulated probabilities of a correct negative decision [P(Correct stop)], incorrect negative decision [P(Incorrect stop)], correct positive decision [P(Correct go)] and incorrect positive decision [P(Incorrect go)] yielding to a total positive [P(Go)] and negative [P(Stop)] decision for both approaches (IRM and MMRM). These probabilities were obtained based on a mixture distribution (reference distribution – purple), where the true treatment effect (Δ_T_) is 0 for 80% of the distribution and Δ_T_ follows a normal distribution centred to a target value (TV) of -2 for both EXACT and E-RS:COPD, -1 for RS-Breathlessness and -0.7 for both RS-Cough & Sputum and RS-Chest Symptoms in the remaining 20% of the distribution.


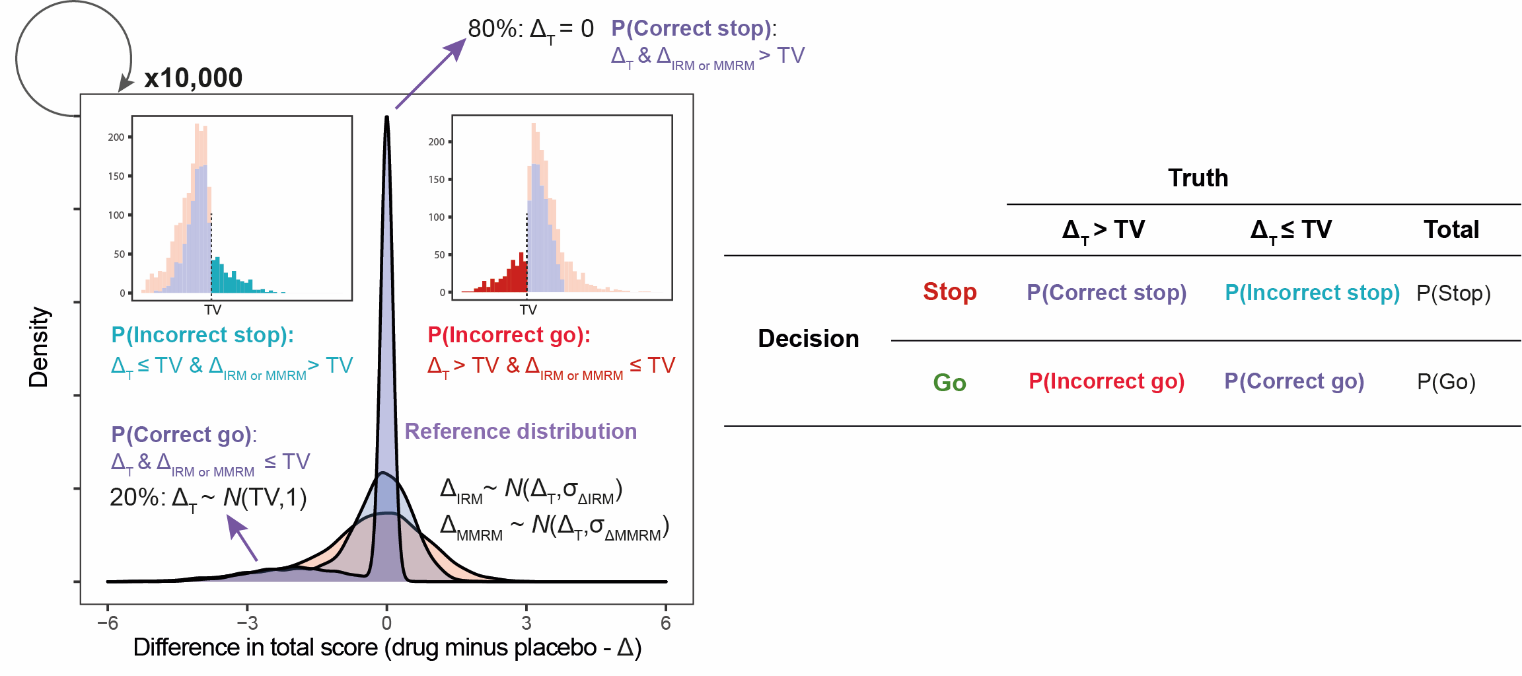


## Fig S4. EXACT score data for each item over 52 weeks of treatment. Each panel represent the item data for drug and placebo arm.


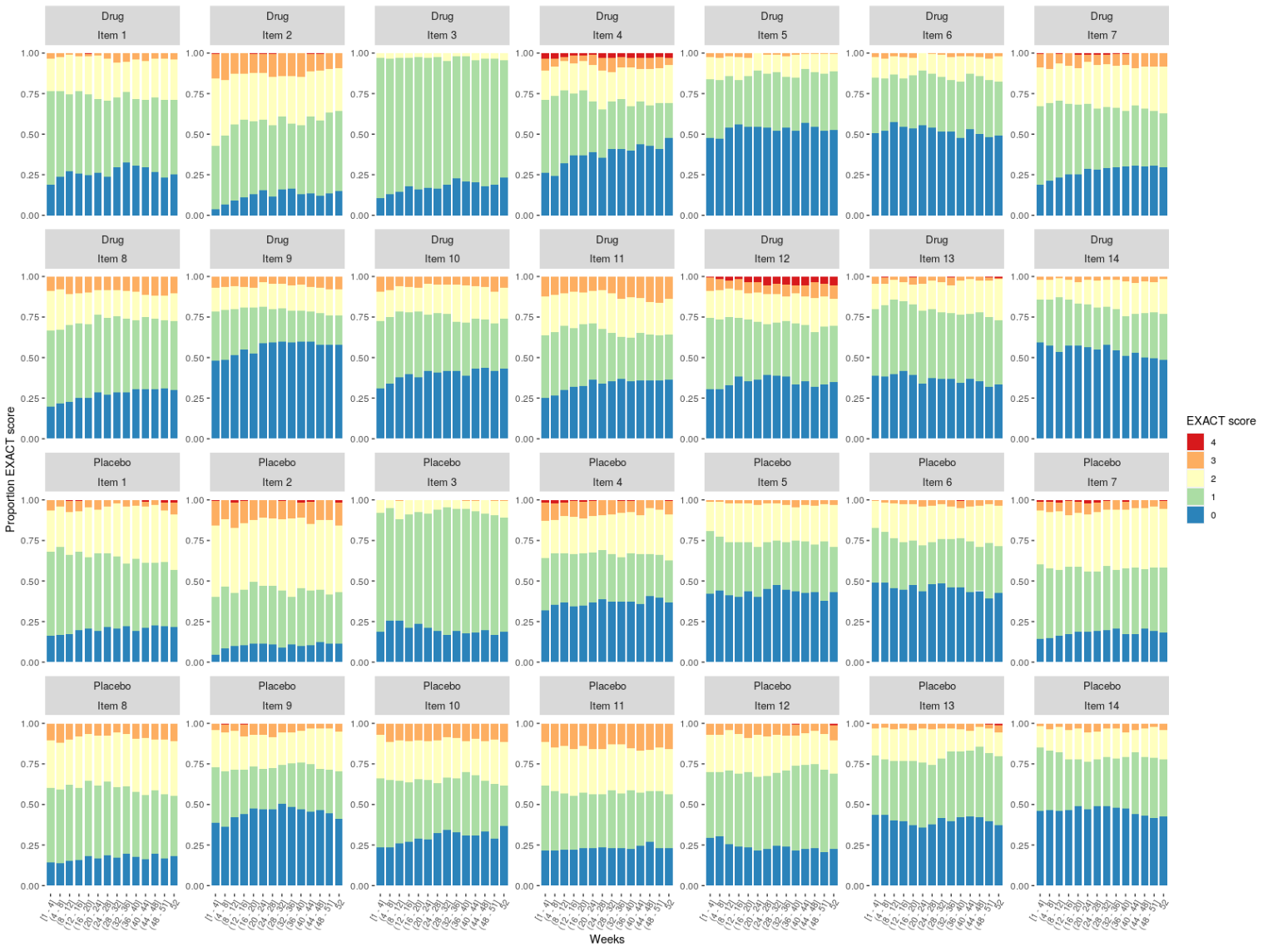


## Fig S5. Item Characteristic curves for all 14 items showing the relationship between patient’s disease status (PSI) and probability of giving a certain score.


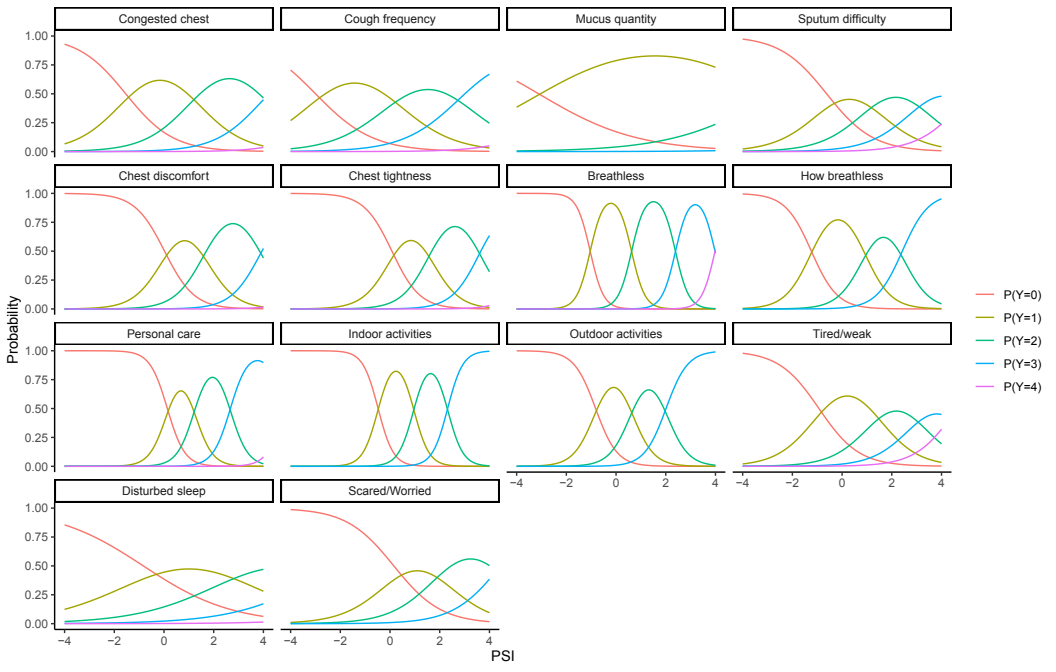


## Fig.S6a. Visual predictive check for the item scores in the treatment arm, stratified by items 1 – 5. Black lines correspond to different proportion of observations and grey areas are the 95% confidence intervals (500 simulations)


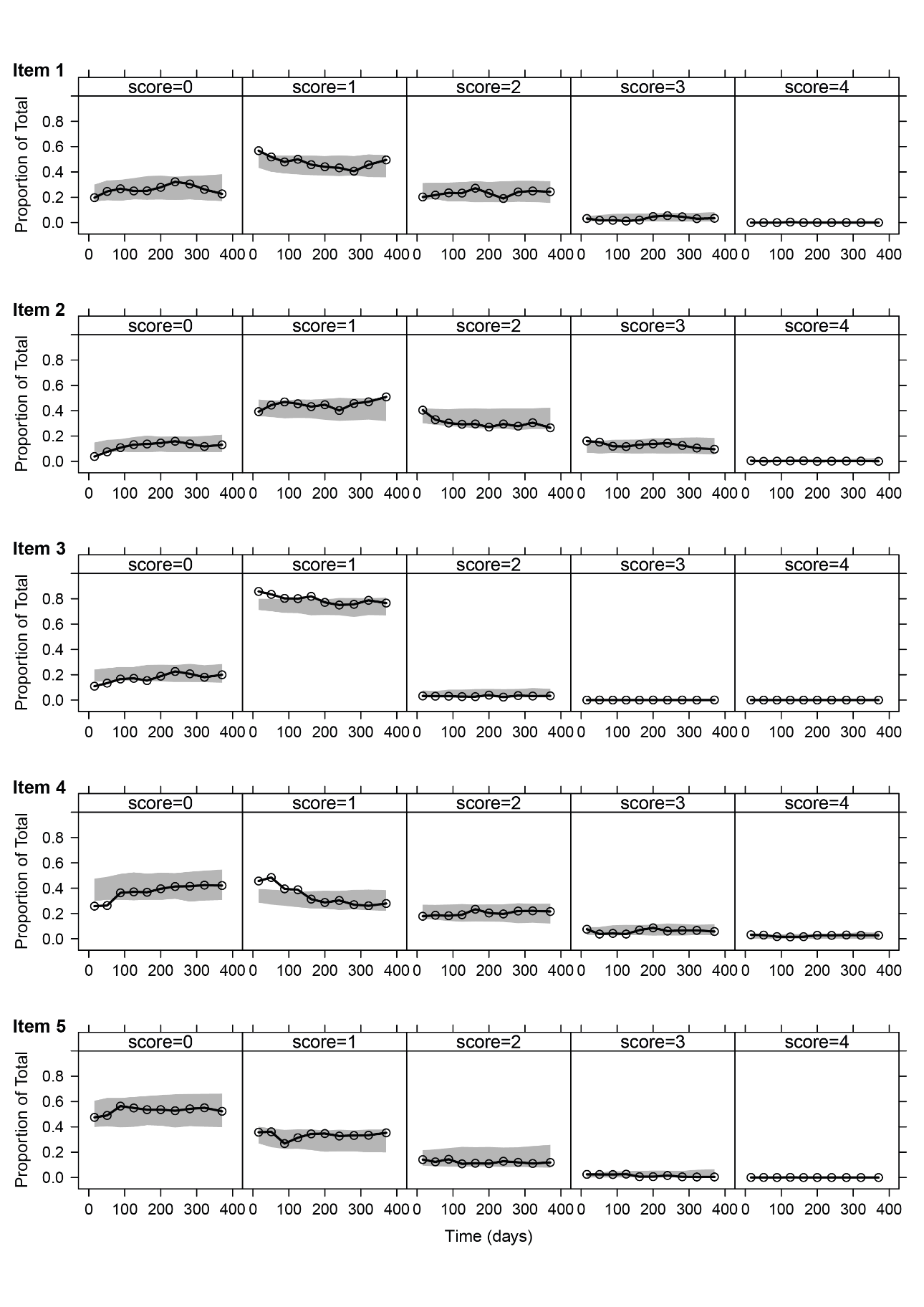


## Fig.S6b. Visual predictive check for the item scores in the treatment arm, stratified by items 6 – 10. Black lines correspond to different proportion of observations and grey areas are the 95% confidence intervals (500 simulations)


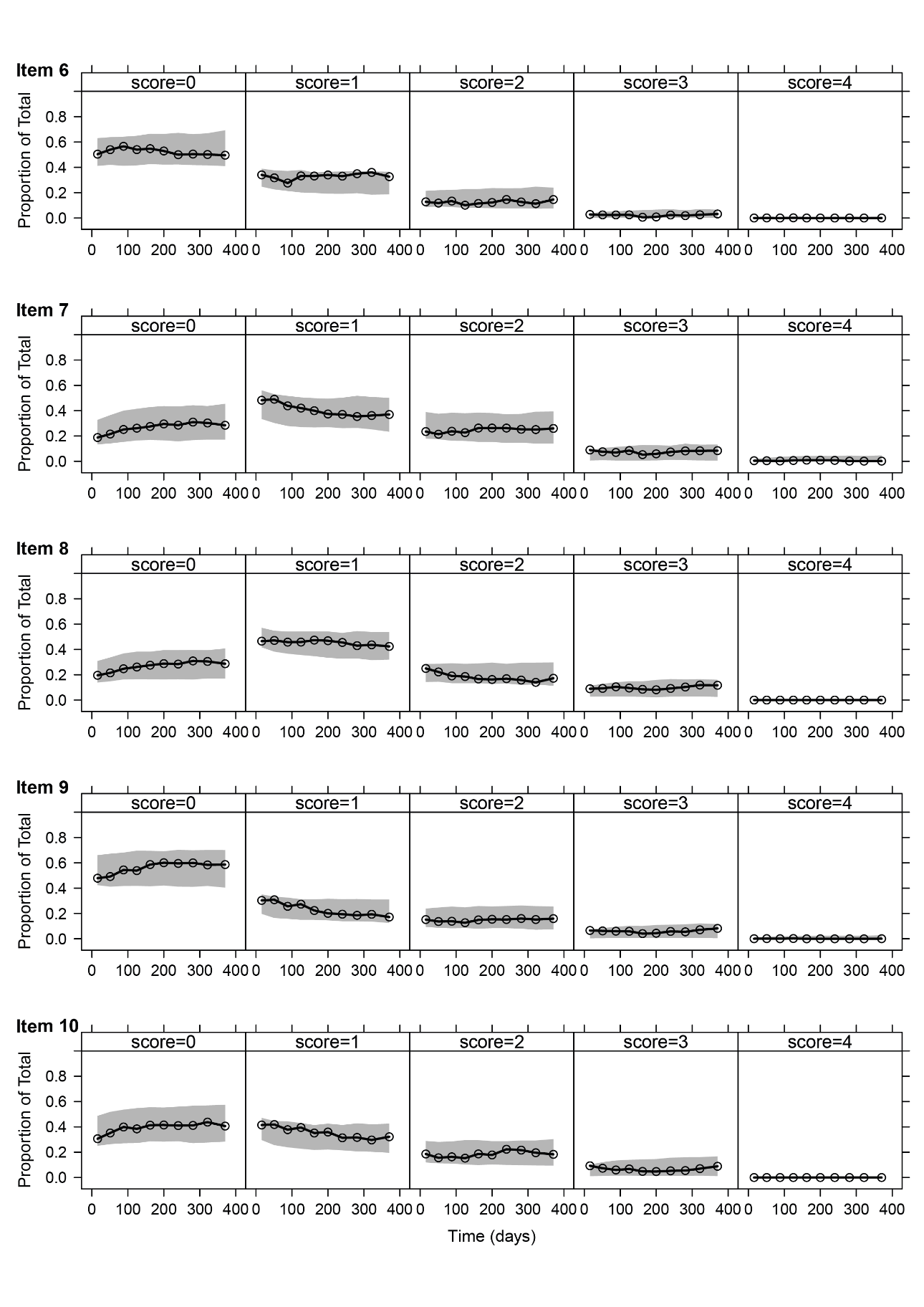


## Fig.S6c. Visual predictive check for the item scores in the treatment arm, stratified by items 11 – 14. Black lines correspond to different proportion of observations and grey areas are the 95% confidence intervals (500 simulations)


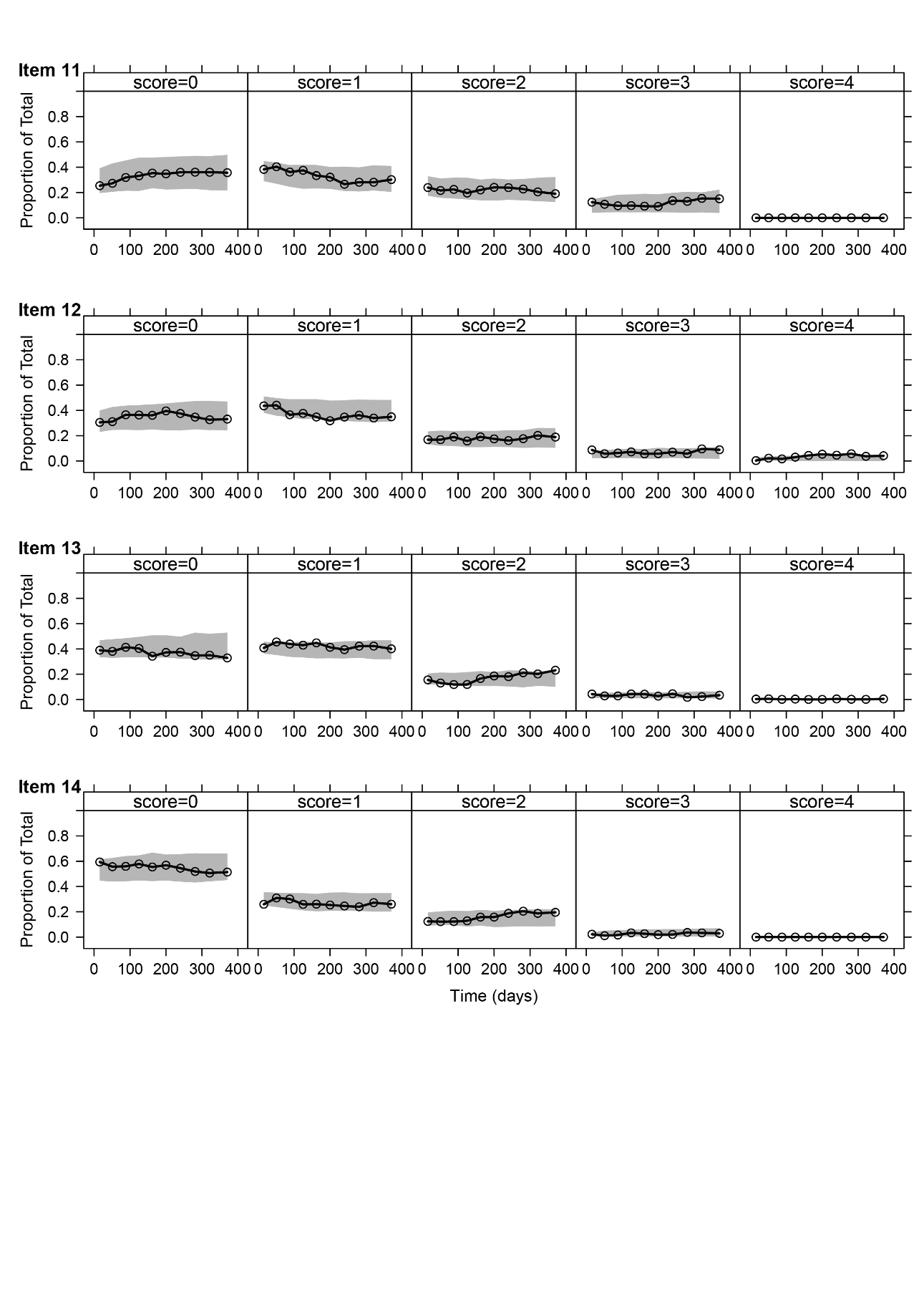


## Fig.S6d. Visual predictive check for the item scores in the placebo arm, stratified by items 1 – 5. Black lines correspond to different proportion of observations and grey areas are the 95% confidence intervals (500 simulations)


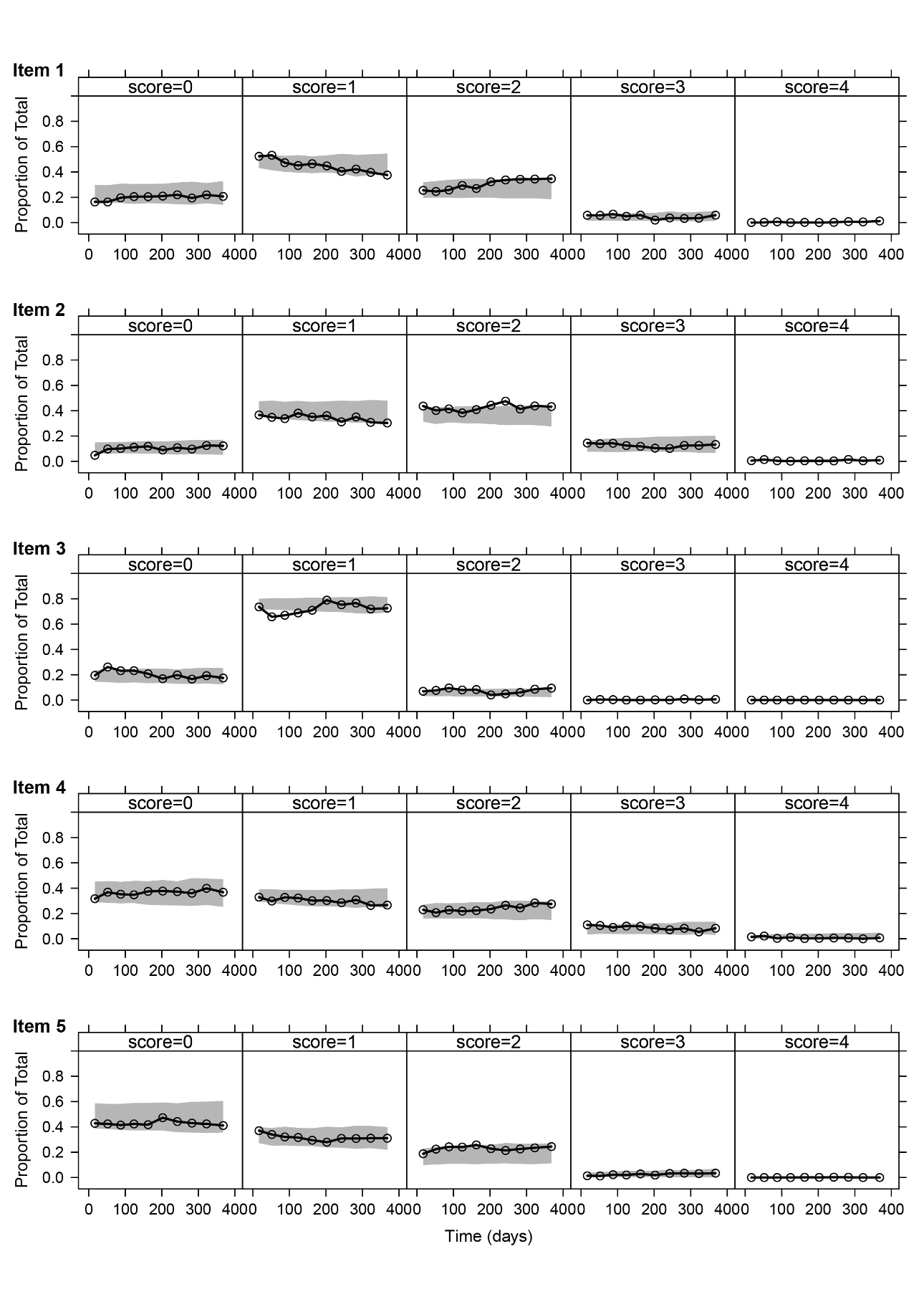


## Fig.S6e. Visual predictive check for the item scores in the placebo arm, stratified by items 6 – 10. Black lines correspond to different proportion of observations and grey areas are the 95% confidence intervals (500 simulations)


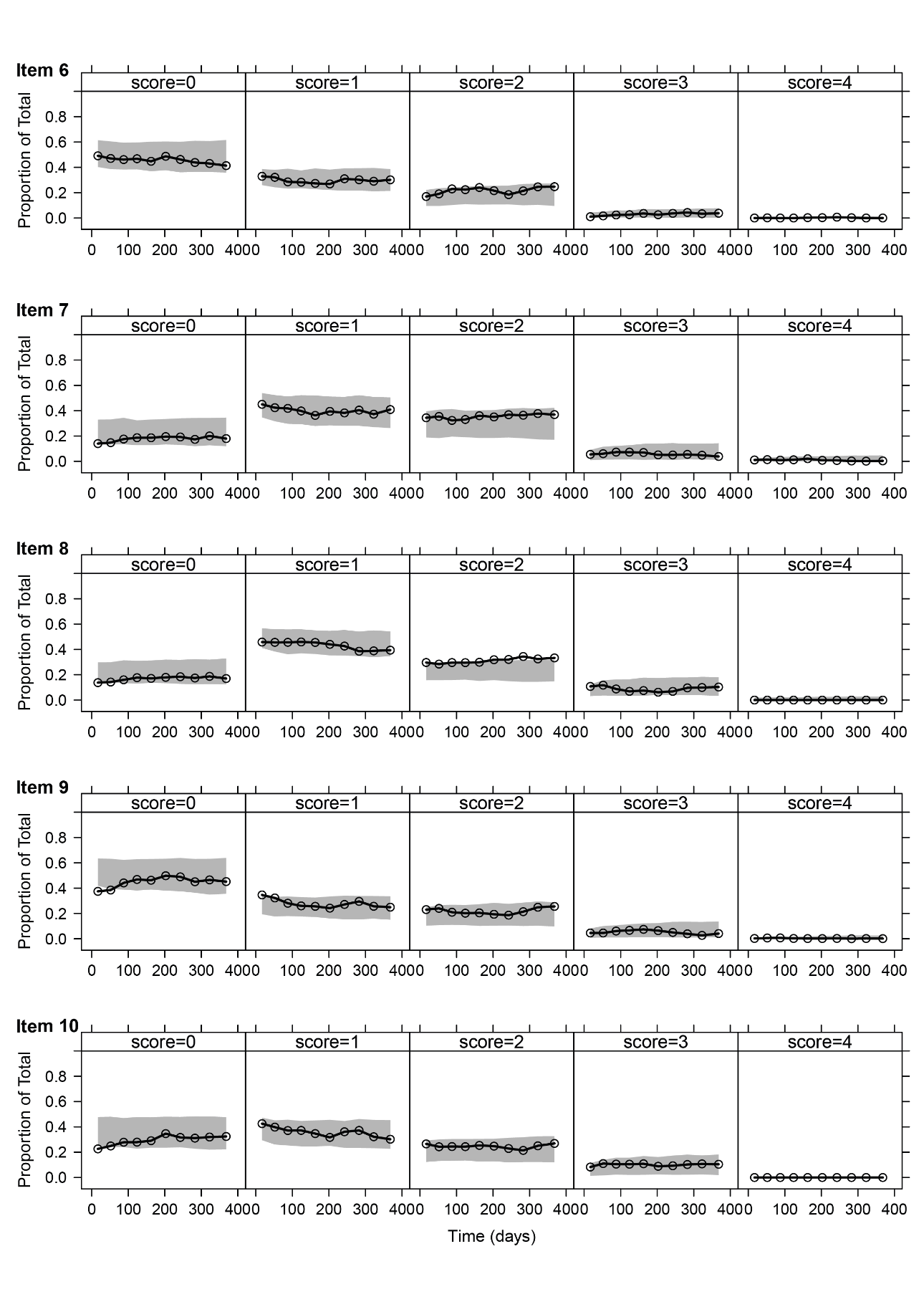


## Fig.S6f. Visual predictive check for the item scores in the placebo arm, stratified by items 11 – 14. Black lines correspond to different proportion of observations and grey areas are the 95% confidence intervals (500 simulations)


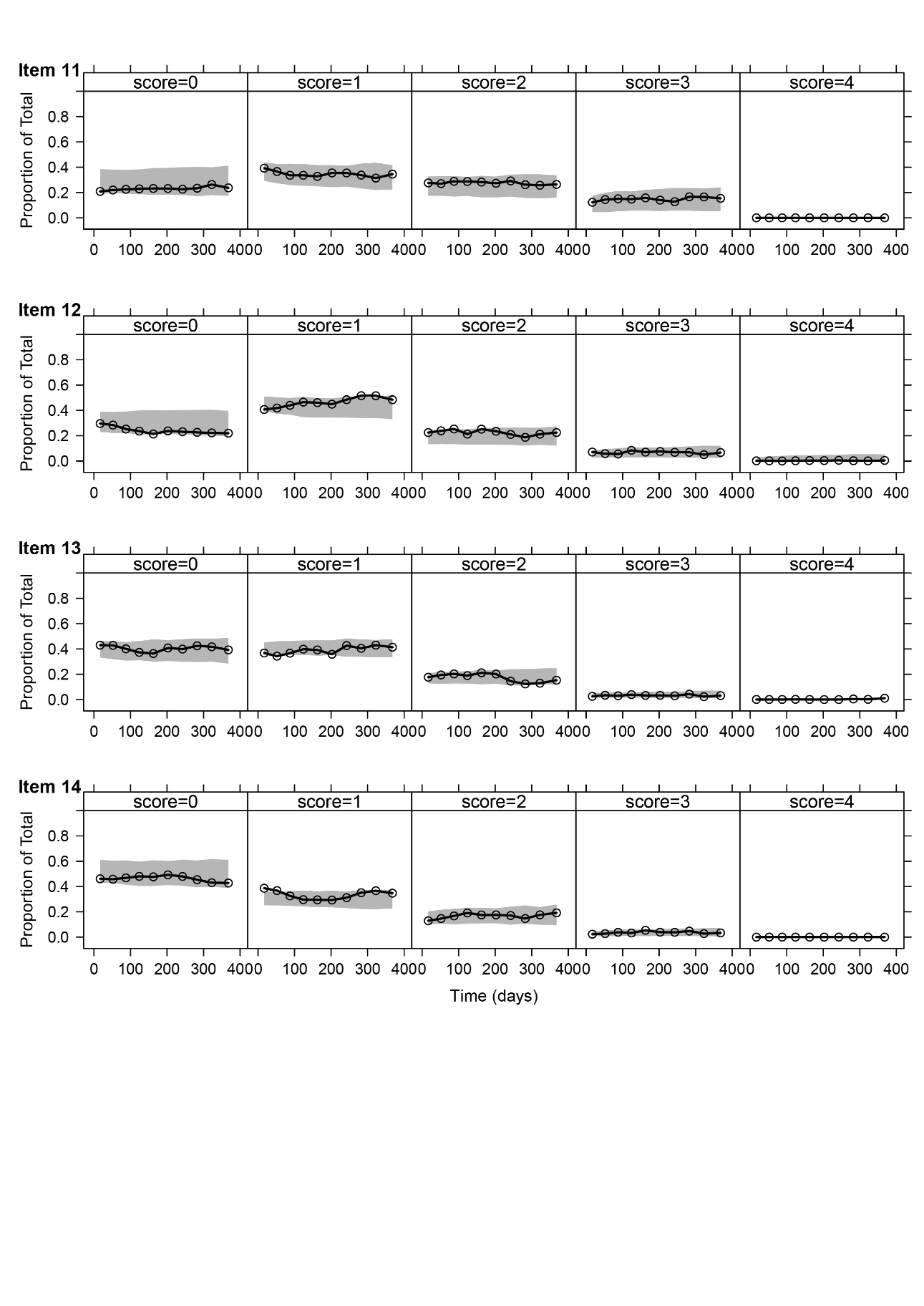


## Fig.S7. Visual predictive check for all 14 items showing proportion of observed transition for treatment (a) and placebo (b) arms. Transitions are described in the panels (black lines) with the corresponding 95% confidence intervals (grey area) from 500 simulations.


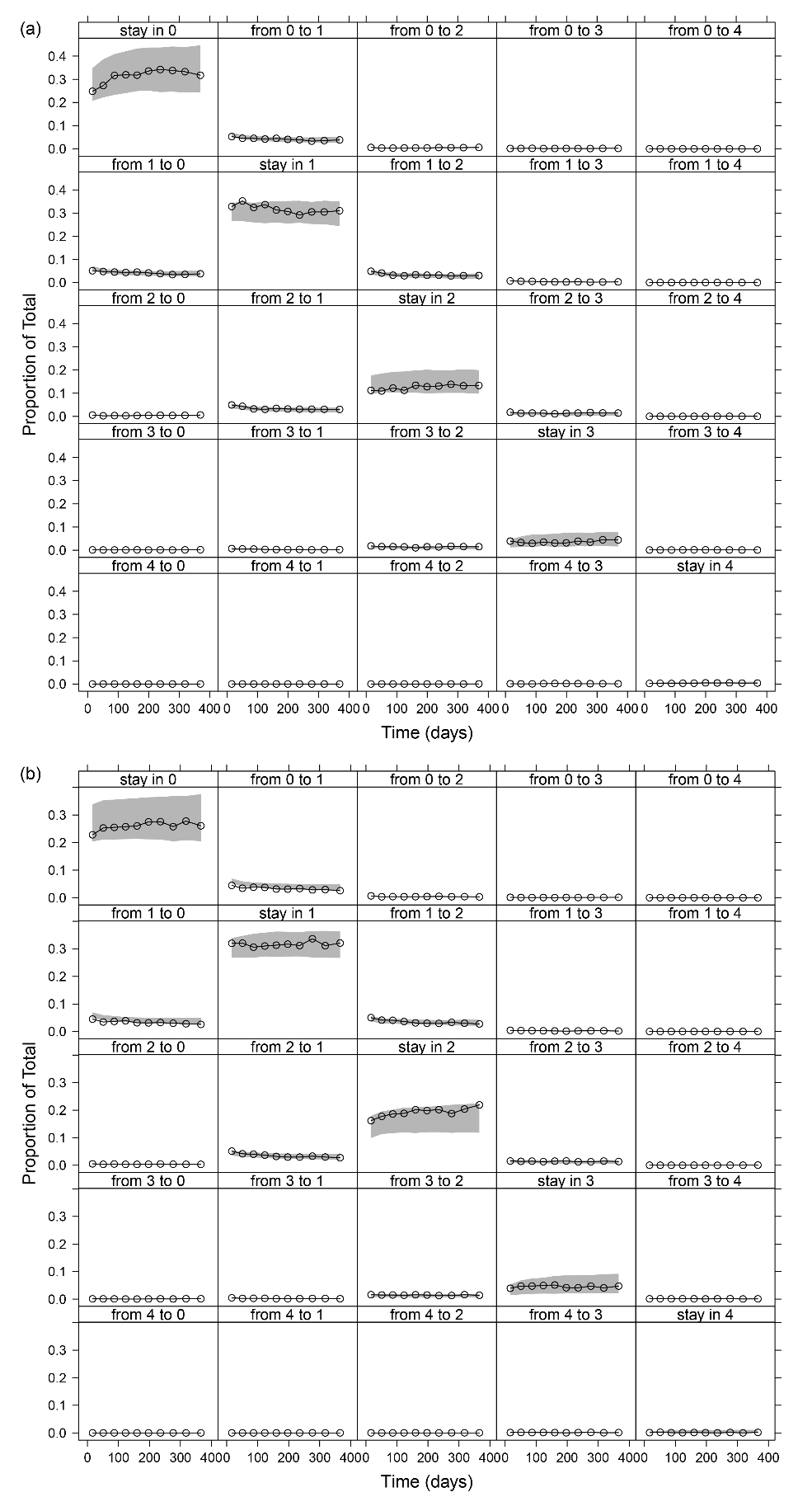


## Fig.S8. ROC curves for the IRM and MMRM analysis considering RS-Breathlessness, RS-Cough and Sputum and RS-Chest Symptoms subscales. AUC-ROC corresponds to the area under the ROC curve and curve and the grey area corresponds to the 95%CI of the ROC curve.


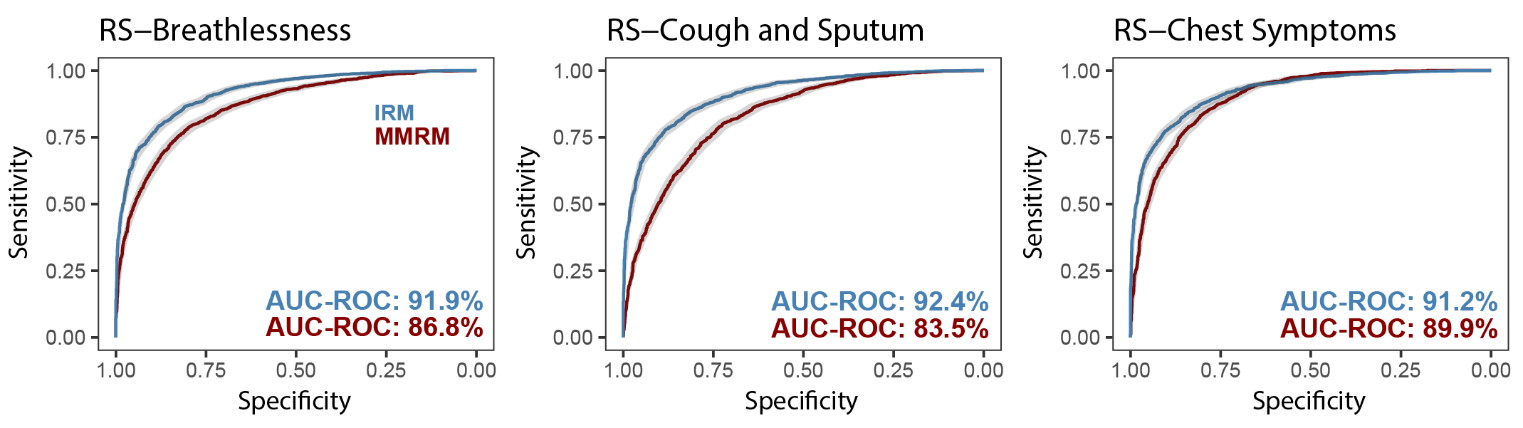

Supplement: Supplementary file 1 — (DOCX 11838 kb) [file 12248_2021_600_MOESM1_ESM.docx]
